# A cold-inducible RNA-binding protein (CIRP)-derived peptide attenuates inflammation and organ injury in septic mice

**DOI:** 10.1038/s41598-017-13139-z

**Published:** 2018-02-12

**Authors:** Fangming Zhang, Max Brenner, Weng-Lang Yang, Ping Wang

**Affiliations:** 1 Center for Immunology and Inflammation, The Feinstein Institute for Medical Research, Manhasset, NY 11030 United States; 2 Department of Surgery, Donald and Barbara Zucker School of Medicine at Hofstra/Northwell, Manhasset, NY 11030 United States

## Abstract

Cold-inducible RNA-binding protein (CIRP) is a novel sepsis inflammatory mediator and C23 is a putative CIRP competitive inhibitor. Therefore, we hypothesized that C23 can ameliorate sepsis-associated injury to the lungs and kidneys. First, we confirmed that C23 dose-dependently inhibited TNF-α release, IκBα degradation, and NF-κB nuclear translocation in macrophages stimulated with CIRP. Next, we observed that male C57BL/6 mice treated with C23 (8 mg/kg BW) at 2 h after cecal ligation and puncture (CLP) had lower serum levels of LDH, ALT, IL-6, TNF-α, and IL-1β (reduced by ≥39%) at 20 h after CLP compared with mice treated with vehicle. C23-treated mice also had improved lung histology, less TUNEL-positive cells, lower serum levels of creatinine (34%) and BUN (26%), and lower kidney expression of NGAL (50%) and KIM-1 (86%). C23-treated mice also had reduced lung and kidney levels of IL-6, TNF-α, and IL-1β. E-selectin and ICAM-1 mRNA was significantly lower in C23-treated mice. The 10-day survival after CLP of vehicle-treated mice was 55%, while that of C23-treated mice was 85%. In summary, C23 decreased systemic, lung, and kidney injury and inflammation, and improved the survival rate after CLP, suggesting that it may be developed as a new treatment for sepsis.

## Introduction

Sepsis is a life-threatening organ dysfunction caused by a dysregulated host response to infection^1–3^. Worldwide, sepsis has been estimated to annually affect 31.5 million individuals and cause 5.3 million deaths^4^. In the US alone, sepsis has an incidence of more than a million new cases per year and accounts for 20% of all admissions to intensive care units^5,6^. Injuries to the lungs and kidneys, in particular, are strong independent contributors to sepsis mortality^7,8^. Yet, despite advances in antibiotic therapy and intensive supportive care, sepsis remains the second leading cause of deaths in non-coronary intensive care units^9^, with an overall mortality rate of up to 30% and accounting for more than 50% of all hospital deaths^10^. The only FDA-approved anti-sepsis medicine, activated protein C (Xigris), was withdrawn from the market by Eli Lilly in 2011 due to limited efficacy and hemorrhagic complications^11^. As a result, no approved effective anti-sepsis pharmacotherapy is available for use in septic patients.

Septic organ failure develops, in large part, due to low oxygen delivery associated with the hypoperfusion caused by vasomotor dysfunction, ventricular dysfunction, and enhanced adrenergic tone^12^. The septic stress not only disrupts crucial cellular and tissue functions, but also exacerbates endoplasmic reticulum stress and leads to the release of damage-associated molecular pattern (DAMP) molecules and inflammatory mediators, which cause leukocyte and vascular endothelial cell (EC) activation, increased capillary permeability, neutrophil infiltration, and tissue injury^12,13^. Cold-inducible RNA-binding protein (CIRP) is a highly conserved RNA-binding nuclear protein that is upregulated by hypoxia, mild hypothermia, and oxidative stress^14–16^. In these conditions, which typically occur during sepsis and shock, CIRP migrates from the nucleus to cytoplasmic stress granules, where it acts as a translational regulator for the messenger RNAs of numerous genes^16,17^. We have discovered that, during sepsis and shock, CIRP not only translocates from the nucleus to the cytoplasm, but it is also subsequently released into the circulation^18^. Once released, CIRP acts as a damage-associated molecular pattern molecule (DAMP) to increase sepsis severity and mortality rate^18,19^. Furthermore, we have also shown that healthy mice injected with CIRP undergo lung vascular endothelial cell (EC) activation, inflammasome activation, and pyroptosis to develop a sepsis-like form of acute lung injury (ALI)^20^. EC activation leads to a loss of endothelial barrier function, increased leukocyte adhesion, a procoagulation state, and vasodilation which, in turn, promote edema, leukocyte infiltration, microcirculatory abnormalities, and distributive shock, all of which aggravate sepsis severity^21^. Furthermore, we have shown that CIRP plays a key role in acute kidney injury (AKI) after renal ischemia and reperfusion^22^. These observations suggest CIRP may be a critical mediator for the development of sepsis-associated organ injury.

In order to identify potential CIRP antagonists, we have screened 32 overlapping 15-mer oligopeptides covering the entire sequence of human CIRP, and identified C23 as a potential CIRP antagonist^18^. C23’s affinity for the TLR4-MD2 receptor complex is one order of magnitude higher than that of CIRP, and two orders of magnitude higher than those of LPS and HMGB1^23,24^. As such, we hypothesized that, by blocking the interaction of extracellular CIRP with its receptor, treatment with C23 should decrease the activation of leukocytes and EC and, thus, attenuate sepsis severity and mortality. Therefore, in this study, we sought to determine the effects of C23 on inflammation and tissue injury in a mouse model of abdominal polymicrobial sepsis.

## Methods

### TNF-α release studies

Rat peritoneal macrophages were collected via peritoneal lavage with 10 ml PBS (Thermo Fisher Scientific, Waltham, MA). Total peritoneal cells were isolated by centrifugation at 200 *g* for 10 min and subsequently cultured in RPMI 1640 complete medium containing 10% of heat inactivated fetal bovine serum, 2 mM L-glutamine, 50 μg/mL gentamicin, 100 U/ml of penicillin and 100 μg/ml streptomycin (all Thermo Fisher Scientific), and 0.05 mM β-mercaptoethanol (Sigma-Aldrich, Billerica, MA) in a humidified incubator with 5% CO_2_ at 37 °C. The adherent cells were mechanically lifted, resuspended in Opti-MEM reduced serum medium (Thermo Fisher Scientific), and plated at a density of 5 × 10^5^ cells/ml. C23 (GRGFSRGGGDRGYGG; >95% purity) was obtained from GenScript USA Inc. (Piscataway, NJ). Plated peritoneal macrophages were pretreated with medium only or 30–300 ng/ml C23 for 1 h, then incubated in the absence or presence of 300 ng/ml recombinant mouse (rm) CIRP^18^ for 6 h. At 6 h, the supernatant was collected for determination of TNF-α levels by ELISA.

### IκBα degradation and NF-κB nuclear translocation study

Murine macrophage RAW 246.7 cells (TIB-71; American Type Culture Collection, Manassas, VA) were cultured in DMEM complete medium (10% of heat inactivated fetal bovine serum, 2 mM L-glutamine, 50 μg/mL gentamicin, 100 units/ml of penicillin and 100 μg/ml streptomycin, and 0.05 mM β-mercaptoethanol) and stimulated with 300 ng/ml rmCIRP for 4 h in the presence or absence of C23. The stimulated RAW cells were then fractionated for Western blotting quantification of IκBα (Cat sc-371; Santa Cruz Biotechnology, Dallas, TX) in the cytosolic fraction using GAPDH (Cat sc-25778; Santa Cruz Biotechnology) as a loading control, and of NF-κB p65 (Cat sc-372; Santa Cruz Biotechnology) in the nuclear fraction using histone (Cat #9715; Cell Signaling, Danvers, MA) as a loading control. Briefly, cell fractions were resolved on NuPAGE 4–12% Bis-Tris protein gels and transferred to nitrocellulose membranes (Thermo Fisher Scientific). After blocking with 5% fat-free powdered milk, the membranes were first incubated with the primary antibodies, and then with goat anti-rabbit secondary detection antibodies (Cat #31460; Thermo Fisher Scientific). The signal was developed with ECL Plus Western blotting substrate (Thermo Fisher Scientific) and analyzed using ImageJ software.

### Animals

Adult C57BL/6 mice (25–30 g) were purchased from Jackson Laboratories (Bar Harbor, ME). Mice present sex dimorphism in their susceptibility and inflammatory response to sepsis^25–28^. Therefore, only male mice were included in this study. Mice were housed in temperature controlled rooms with 12-h light cycles, fed a standard mouse diet, and allowed to acclimate for one week before experimentation. All experiments involving live animals were carried out in agreement with the National Institutes of Health guidelines for the use of experimental animals and were reviewed and approved by the Institutional Animal Care and Use Committee at the Feinstein Institute for Medical Research.

### Animal model of polymicrobial abdominal sepsis

Mice were anesthetized with isoflurane and placed in the supine position. The ventral abdomen was then shaved and cleaned with 10% povidone-iodine alternated three times with 70% alcohol followed by a final application of disinfectant solution. Cecal ligation and puncture (CLP) was performed as described previously^18^. Briefly, a 2-cm midline laparotomy was performed. The cecum was exposed, ligated with a 4–0 silk suture 0.75–1 cm proximal from the distal cecal extremity, and punctured twice with a 22-gauge needle. A small amount of cecal content was then extruded from the perforation sites, and the ligated cecum was returned to the peritoneal cavity. The laparotomy wound was suture-closed with 4–0 silk in two layers. Sham operated animals underwent the same procedure, except that the cecum was neither ligated nor punctured. Immediately after surgery, animals received a subcutaneous injection of 1 ml normal saline. After recovery from anesthesia, mice were returned to their cages. Mice were not treated with opioids to avoid lowering their respiratory drive, were not treated with non-steroidal anti-inflammatory drugs to avoid acute kidney injury and digestive tract ulcers. Antibiotics were not used in order for the mice to develop severe sepsis with early mortality. At 20 h after CLP, mice were euthanized for blood and tissue collection. For the 10-day experiment, mice were evaluated daily for their survival status. Sham-operated mice were not expected to die within the observation period and thus were not included in the survival experiment.

### Treatment with vehicle and C23

At 2 h after CLP, each mouse was randomly allocated to receive a 200-μl jugular vein injection containing vehicle (normal saline solution) or 8 mg/kg BW C23. C23’s dose of administration was selected based on our previous experience using C23 to treat a mouse model of hemorrhagic shock^18,29^.

### Collection of blood and tissues

At 20 h after CLP, mice were anesthetized with isoflurane for blood and tissue collection. After clotting, blood samples were centrifuged at 1000 *g* for 10 min at 4 °C, and the resulting serum samples were stored at −80 °C until assayed. Upper and lower lobe lung and kidney samples were preserved in 10% formalin for histopathological analysis. The remaining tissue was flash-frozen in liquid nitrogen and stored at −80 °C until assayed.

### Determination of organ injury marker and cytokine levels

Serum levels of alanine aminotransferase (ALT), lactate dehydrogenase (LDH), creatinine, and blood urea nitrogen (BUN) were determined using colorimetric assays (Pointe Scientific, Canton, MI). Serum and tissue levels of interleukin 6 (IL-6), interleukin 1β (IL-1β), and tumor necrosis factor α (TNF-α) were determined using mouse-specific ELISA kits (BD Biosciences, San Diego, CA). All assays were carried out according to manufacturer’s instructions. Cytokine measurements have larger sample sizes because cytokine levels were also determined in a small replication cohort.

### Histological evaluation of lung injury and TUNEL staining

Formalin-fixed lung samples were embedded in paraffin, microsectioned at 4 μm, and stained with hematoxylin and eosin (HE). Histological examination was conducted by an examiner blinded to treatment allocation. Lung injury was assessed using a previously described semi-quantitative light microscopy scoring system^30^. Histological scores have larger sample sizes because they were also determined in a small replication cohort. For terminal deoxynucleotidyl transferase dUTP nick-end labeling (TUNEL) staining, fluorescence staining was performed using a commercially available *in situ* Cell Death Detection Kit (Roche Diagnostics, Indianapolis, IN). The assay was conducted according to the manufacturer’s instructions. Results are expressed as the average number of TUNEL-positive staining cells per 200× magnification field.

### Quantification of NGAL, KIM-1, E-selectin, and ICAM1 mRNA

The renal mRNA expression of neutrophil gelatinase-associated lipocalin (NGAL) and kidney injury molecule-1 (KIM-1) and pulmonary expression of E-selectin and intercellular adhesion molecule 1 (ICAM1) were assessed by real-time quantitative PCR (qPCR). After each cryopreserved tissue was crushed, the total RNA was extracted using TRIzol reagent (Invitrogen, Carlsbad, CA). The RNA was then reverse-transcribed into cDNA using murine leukemia virus reverse transcriptase (ThermoFisher Scientific). qPCR reactions were carried out in 25 μl containing 0.08 μmol of each forward and reverse primer (Table 1), 5 μl cDNA, 6.5 μl H_2_O, and 12.5 μl SYBR Green PCR Master Mix (ThermoFisher Scientific). Amplification was conducted in duplicates in a 7300 real-time thermocycler (Applied Biosystems, Foster City, CA) with a thermal profile of 50 °C for 2 min and 95 °C for 10 min, followed by 45 cycles of 95 °C for 15 s and 60 °C for 1 min. Relative expression of mRNA normalized to mouse β-actin was determined using the 2^(−ΔΔ*Ct*)^ method^31^.

**Table 1:** Genes and primers used for mRNA amplification.

Protein	Gene	RefSeq	Forward primer	Reverse primer
β-actin	*Actb*	NM_007393	CGTGAAAAGATGACCCAGATCA	TGGTACGACCAGAGGCATACAG
NGAL	*Lcn2*	NM_005564.4	CTCAGAACTTGATCCCTGCC	TCCTTGAGGCCCAGAGACTT
KIM-1	*Havcr1*	NM_134248.2	TGCTGCTACTGCTCCTTGTG	GGGCCACTGGTACTCATTCT

### Statistical analysis

Results are reported as least square mean ± SEM. Sample sizes assumed a two-tailed statistical significance of 0.05 or less and a power of 0.8. Sample sizes for the sham group included a smaller number of animals because measurements in this group are significantly more homogeneous. All normality distributed data was expressed as mean ± standard error of the mean (SE) and compared using analysis of variance (ANOVA) with Student-Newman-Keul’s post-hoc analysis, with WT sham as the control group. Non-normally distributed data were compared using ANOVA on Ranks. Differences in values were considered significant if *p* < 0.05. All statistical analyses were performed using SigmaStat (Systat Software Inc, Chicago, IL). Due to statistical power limitations, differences between groups that were not statistically significant were considered non-informative.

### Ethics approval

All experiments involving live animals were carried out in accordance with the National Institutes of Health guidelines for the use of experimental animals and were reviewed and approved by the Institutional Animal Care and Use Committee at the Feinstein Institute for Medical Research.

### Availability of data and materials

The datasets used and analyzed during the current study are available from the corresponding author upon reasonable request.

## Results

### C23 dose-dependently inhibits CIRP-induced macrophage activation

**Figure 1:**
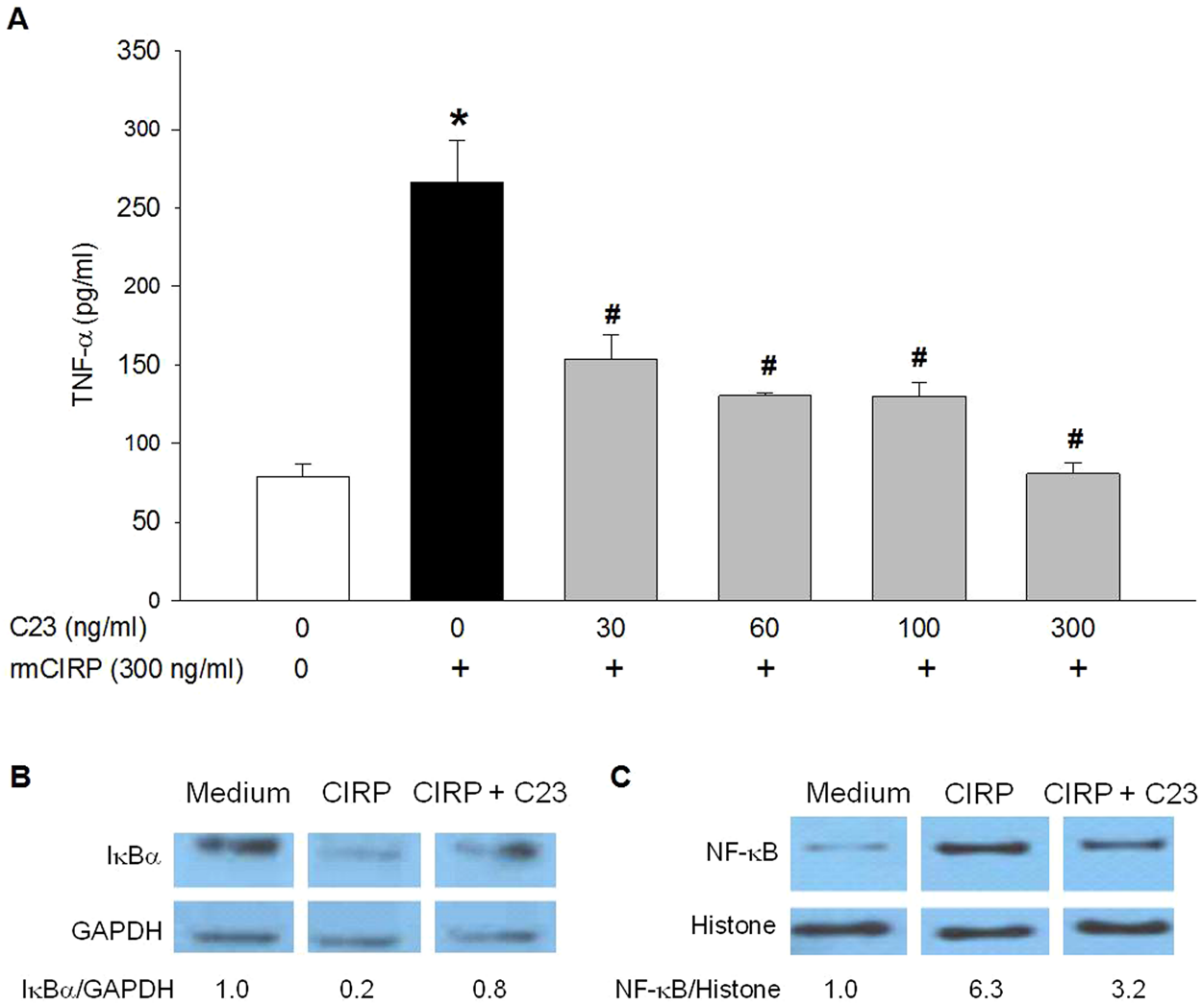
C23 inhibits CIRP-induced macrophage activation. (**A**) C23 dose-dependently decreased rmCIRP-induced TNF-α production in rat peritoneal macrophages. Cells were pretreated with the indicated concentrations of C23 for 1 h and then stimulated with rmCIRP for 6 h. *Data are presented as means* ± *SE*. *Medium alone* = *8* *replicates; rmCIRP alone* = *10* *replicates; C23* = *3* *replicates per group*. *p < *0.05* *vs*. *medium control*, ^#^*p* < *0.05* *vs*. *rmCIRP alone; one-way ANOVA followed by Student-Newman-Keuls test*. (**B**) rmCIRP promoted degradation of cytosolic IκBα in cultured RAW 264.7 cells, which was partially reverted by C23. Cells were co-incubated with rmCIRP (300ng/ml) in the absence or presence of C23 for 4 h, fractionated, and immunoblotted using antibodies for IκBα and GAPDH. The indicated values represent the ratio between IκBα and GAPDH, relative to the medium control. (**C**) rmCIRP promoted nuclear translocation of NF-κB p65 in cultured RAW 264.7 cells, which was partially reverted by C23. Nuclear extracts were immunoblotted using antibodies for NF-κB p65 and histone. The indicated values represent the ratio between NF-κB and histone, relative to the medium control. The blots in (**B**) and (**C**) consist of different parts of the same gel, for a single sample; the full blots are included in the Supplementary Information.

We have shown that CIRP induces macrophage release of TNF-α^18^. To assess C23’s ability to inhibit CIRP-induced macrophage release of TNF-α, we pre-incubated rat peritoneal macrophages with culture medium only or various concentrations of C23, stimulated the cells with rmCIRP, and measured TNF-α in the supernatant. C23 dose-dependently inhibited TNF-α release after macrophage stimulation with rmCIRP (Fig. 1A).

In a different experiment, we incubated RAW 264.7 cells with medium, rmCIRP, or rmCIRP plus C23 for 4 h. We subsequently collected the cells, obtained cytosolic and nuclear fractions, and measured IκBα and NF-κB by Western blotting. In the cytosolic fraction, stimulation with rmCIRP induced IκBα degradation, as indicated by lower IκBα levels, which was reverted by co-incubation with C23 (Fig. 1B). In the nuclear fraction, stimulation with rmCIRP induced NF-κB nuclear translocation, as indicated by increased levels of NF-κB, which was partially reverted by co-incubation with C23 (Fig. 1C). Taken together, these results demonstrate that C23 inhibits CIRP activation of macrophages resulting in reduced IκBα degradation, decreased NF-κB nuclear translocation, and decreased TNF-α release.

### C23 decreases systemic proinflammatory cytokines and organ injury markers after CLP

*In vivo*, CIRP promotes the release of proinflammatory cytokines^18,20^. To evaluate the effects of C23 on systemic inflammation, we measured the level of proinflammatory cytokines in sera collected 20 h after CLP. The serum levels of IL-6, TNF-α, and IL-1β were significantly higher (958.2-, 124.6-, and 6.9-fold, respectively) in vehicle-treated mice than in sham-operated mice (Table 2). Nonetheless, TNF-α and IL-1β levels were significantly lower (57% and 46% lower, respectively) in C23-treated mice than in vehicle-treated mice (Table 2). Likewise, there was a statistical trend towards lower IL-6 levels in C23-treated mice (Table 2). These results demonstrate that C23 significantly dampens the massive systemic inflammation typical of sepsis.

**Table 2:** Effects of C23 on systemic and tissue levels of proinflammatory cytokines at 20 h after CLP.

Cytokine	Sham	Vehicle	*p*-value	C23	*p*-value	*p*-value
	Mean ± SE	Mean ± SE	vs. sham	Median ± SE	vs. sham	vs. veh
**Serum (pg/ml)**						
IL-6	34.8 ± 16.7	33,364.40 ± 6,312.60	0.001	18,341.10 ± 3.8	0.018	0.059
TNF-α	3.4 ± 0.5	416.5 ± 60.7	<0.001	180.1 ± 16.9	0.032	<0.001
IL-1β	7.8 ± 2	53.5 ± 10.8	0.005	28.7 ± 2.2	0.127	0.028
**Lung (pg/mg)**						
IL-6	187 ± 8.7	2,369.20 ± 481.2	<0.001	1,024.80 ± 240.5	0.044	0.005
TNF-α	67.5±1.7	185.6 ± 11	<0.001	133.5 ± 4.3	<0.001	<0.001
IL-1β	179.9 ± 4	454.3 ± 20.8	<0.001	346 ± 14.7	<0.001	<0.001
**Kidney (pg/mg)**						
IL-6	724.7 ± 20.3	2,075.30 ± 285.3	<0.001	1,220.50 ± 128.6	0.038	0.003
TNF-α	301.5 ± 11.2	676 ± 31.1	<0.001	599.2 ± 35.4	<0.001	0.087
IL-1β	934 ± 8.4	2,383.40 ± 93.3	<0.001	2,167.80 ± 62.7	<0.001	0.051

Note: Number of animals in each group: sham n = 5, vehicle (veh) n = 12, C23 n = 11. One-way ANOVA with Student-Newman-Keuls test.

**Figure 2:**
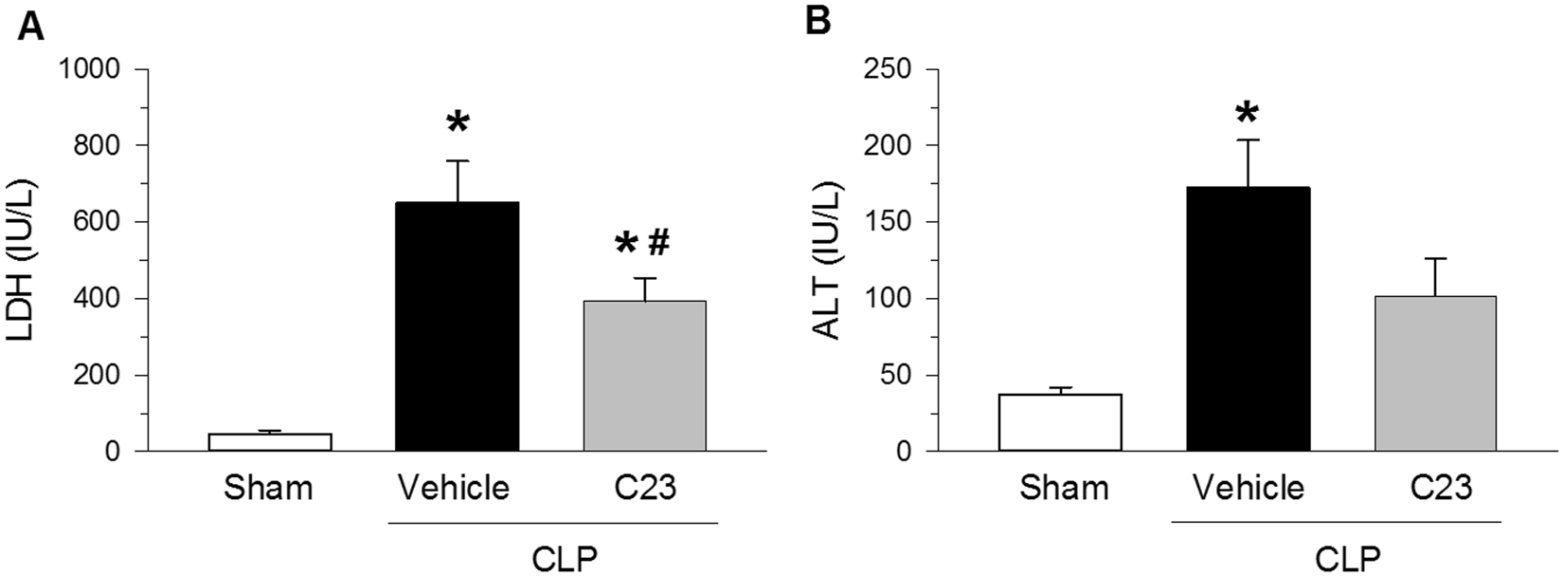
C23 attenuates organ injury after CLP. At 20 h after CLP. (**A**) LDH serum levels were significantly elevated in mice treated with vehicle, and significantly decreased in mice treated with C23. (**B**) ALT serum levels were significantly elevated in mice treated with vehicle, and there was a trend towards lower levels in C23-treated mice (C23 vs. vehicle, *p* = 0.063). *Data are presented as means* ± *SE*. *Number of animals in each group: sham n* = *5*, *vehicle n* = *7*, *C23 n* = *6*. *p < *0.05* *vs*. *sham*, ^#^*p* < *0.05* *vs*. *vehicle; one-way ANOVA followed by Student-Newman-Keuls test*.

LDH and ALT are organ injury markers released mainly by the liver, though LDH can also be released by myocytes, cardiomyocytes, and red blood cells^32^. To determine whether treatment with C23 attenuates systemic organ injury after CLP, we measured the serum levels of LDH and ALT. At 20 h after CLP, LDH and ALT levels were significantly higher (14.1- and 4.6-fold, respectively) in septic mice treated with vehicle than in sham-operated mice (Fig. 2). Compared with vehicle, C23-treated mice had significantly lower serum levels of LDH (39% lower), and also showed a statistical trend towards lower serum levels of ALT (41% lower, Fig. 2). These results indicate that treatment of sepsis with C23 is able to attenuate systemic organ injury.

### C23 attenuates lung injury after CLP

**Figure 3:**
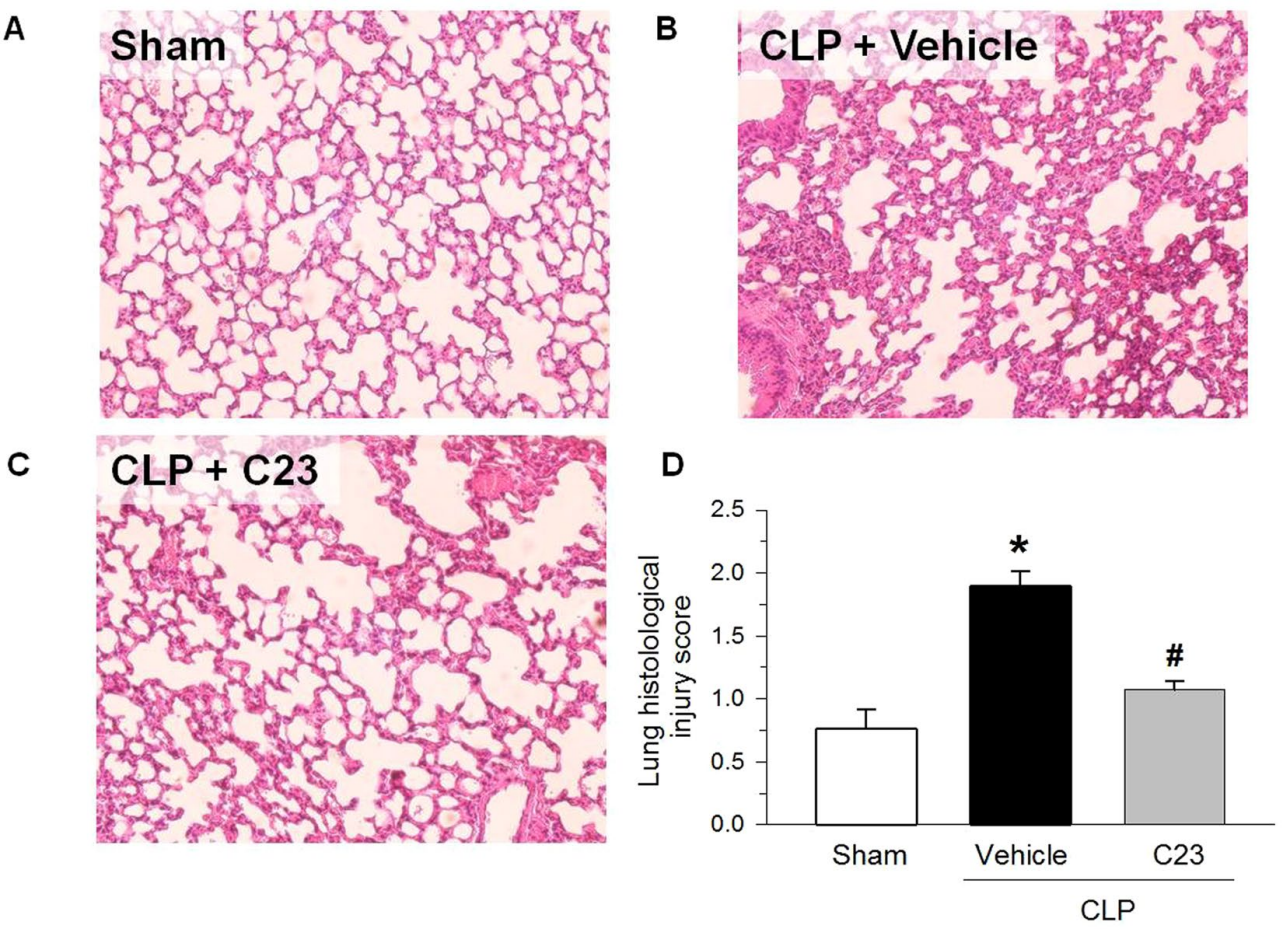
C23 improves lung histological architecture after CLP. At 20 h after CLP, (**A**) the lungs of sham-operated mice showed normal histological architecture, while the lungs of septic mice treated with (**B**) vehicle or (**C**) C23 displayed alveolar wall thickening with predominantly interstitial neutrophil accumulation. *HE staining; representative photomicrographs*, *200*×. (**D**) The lung histological injury score, graded by an investigator blinded to the interventions, was significantly higher in mice adjuvantly treated with vehicle than in mice treated with C23. *Data are presented as means* ± *SE*. *Number of animals in each group: sham n* = *5*, *vehicle n* = *12*, *C23 n* = *11*. *p < *0.05* *vs*. *sham*, ^#^*p* < *0.05* *vs*. *vehicle; one-way ANOVA followed by Student-Newman-Keuls test*.

Sepsis is often associated with ALI, and CIRP injection is sufficient to cause lung injury^20^. To assess C23’s effect on changes in lung morphology induced by sepsis, we examined HE-stained lung sections with light microscopy. At 20 h after surgery, sham-operated mice exhibited normal lung architecture, but the lungs of vehicle-treated CLP mice had alveolar wall thickening and interstitial accumulation of neutrophils (Fig. 3A). However, the lung injury score of mice adjuvantly treated with C23 was significantly reduced by 43% compared with vehicle (Fig. 3B). The improvement in lung alveolar edema and inflammatory infiltrates suggest that C23 is able to attenuate sepsis-induced lung injury.

Infiltrating neutrophils are an important source of proinflammatory cytokines in indirect lung injury. To evaluate the effects of C23 on sepsis-induced inflammation in the lungs, we assessed proinflammatory cytokine levels in lungs collected 20 h after CLP. Similarly to serum levels, the lung levels of IL-6, TNF-α, and IL-1β were significantly higher (12.7-, 2.7-, and 2.5-fold, respectively) in vehicle-treated mice than in sham-operated mice (Table 2). Also similarly to serum levels, lung levels of IL-6, TNF-α, and IL-1β were significantly lower (57%, 28%, and 24% lower, respectively) in C23-treated mice than in vehicle-treated mice (Table 2). These data indicate that C23 is able to decrease local lung inflammation caused by sepsis.

**Figure 4:**
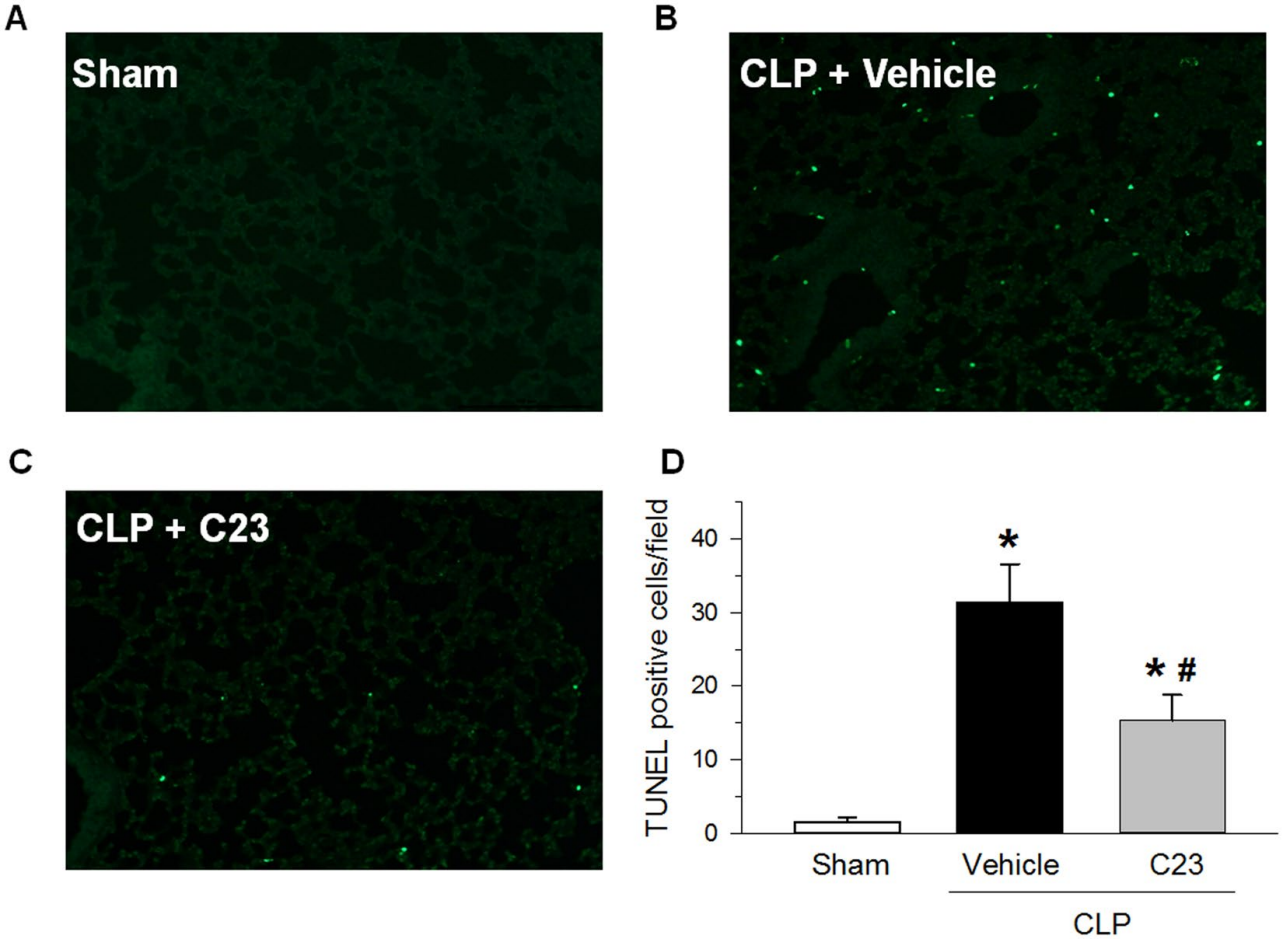
C23 decreases lung TUNEL-positive cells after CLP. At 20 h after CLP, the lungs of sham-operated mice showed rare TUNEL-positive cells. The lungs of septic mice treated with vehicle had a large number of TUNEL-positive cells, while the lungs of septic mice treated with C23 had a significantly reduced number of TUNEL-positive cells. *Representative photomicrographs*, *200* × . *Data are presented as means* ± *SE* (*n* = *3* *per group*). *p < *0.05* *vs*. *sham*, ^#^*p* < *0.05* *vs*. *vehicle; one-way ANOVA followed by Student-Newman-Keuls test*.

We have shown that CIRP promotes apoptosis in the lungs of mice with sepsis^13,20^. To determine whether C23 can decrease lung apoptotic events during sepsis, we quantified the number of TUNEL-positive events in lungs collected 20 h after CLP. As expected, the lungs of vehicle-treated CLP mice had 20.9-times more TUNEL-positive cells than those of sham-operated mice (Fig. 4). The number of TUNEL-positive cells in the lungs of C23-treated CLP mice, however, was 51% lower than that of vehicle-treated mice (Fig. 4). These findings indicate that C23 decreases cell death in the lungs of mice with sepsis.

### C23 decreases kidney endothelial cell activation after CLP

**Figure 5:**
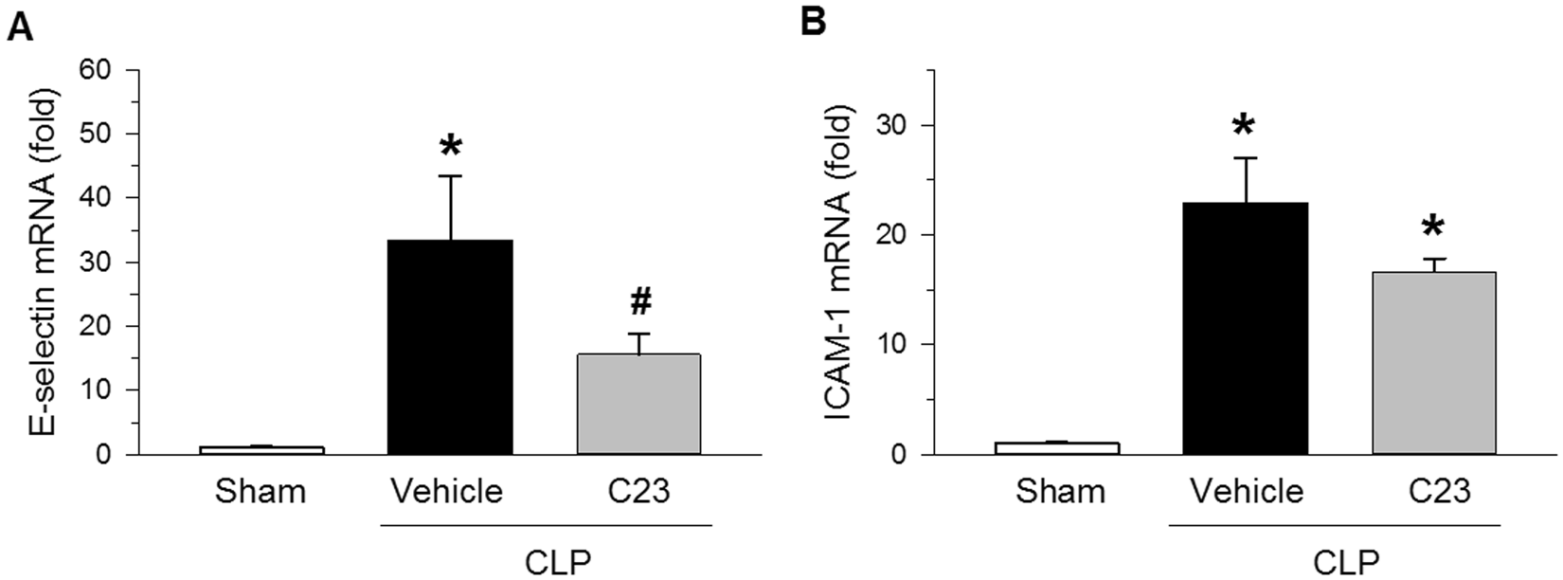
C23 decreases kidney endothelial cell activation after CLP. The mRNA expression of endothelial cell activation markers was quantified in kidney tissue samples collected at 20 h after CLP. (**A**) E-selectin gene expression was significantly elevated in the vehicle group, and significantly reduced in C23-treated mice. (**B**) ICAM-1 gene expression was significantly elevated in the vehicle group, and there was a trend towards lower expression in C23-treated mice (C23 vs. vehicle, *p* = 0.079). *Data are presented as means* ± *SE*. *Number of animals in each group: sham n* = *5*, *vehicle n* = *7*, *C23 n* = *6*. *p < *0.05* *vs*. *sham*, ^#^*p* < *0.05* *vs*. *vehicle; one-way ANOVA followed by Student-Newman-Keuls test*.

We have previously shown that CIRP mediates lung vascular endothelial cell (EC) barrier dysfunction and EC activation^20^ and that adjuvant treatment with C23 attenuates vascular permeability caused by hemorrhagic shock^29^. Therefore, we determined C23’s effect on the mRNA expression of endothelial cell activation markers in kidneys collected 20 h after CLP. Compared with sham-operated mice, the kidneys of vehicle-treated CLP mice expressed significantly higher levels of e-selectin and ICAM-1 (31.2- and 22.5-fold, respectively) (Fig. 5). Conversely, C23-treated mice expressed significantly less E-selectin (53% less), and had a trend towards also expressing less ICAM-1 (27% less) compared with vehicle-treated mice (Fig. 5). These results suggest that C23 reduces EC activation during sepsis.

### C23 attenuates sepsis-associated AKI

**Figure 6:**
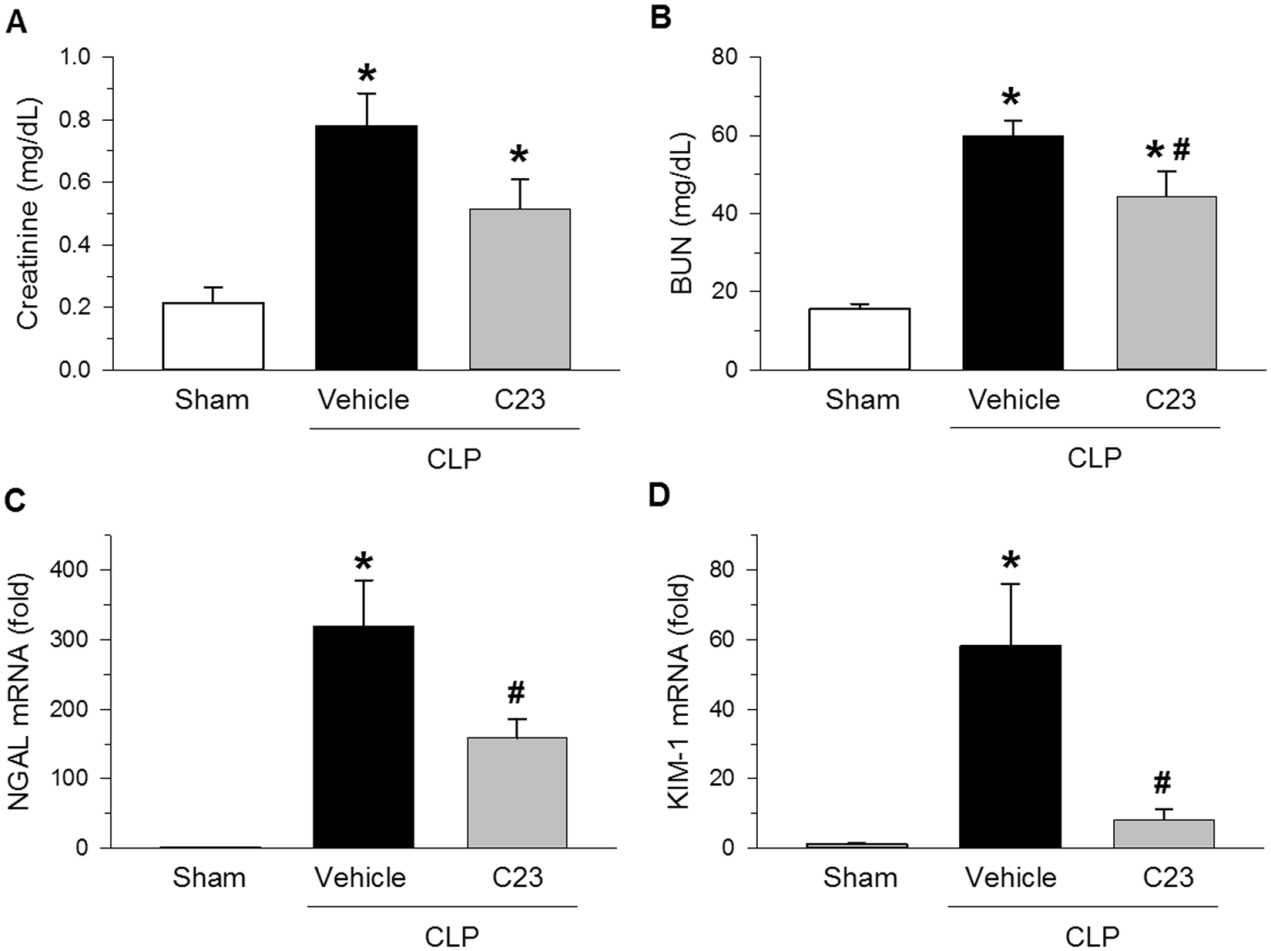
C23 attenuates AKI after CLP. Renal function and injury was evaluated in serum and kidney tissue samples collected at 20 h after CLP. (**A**) The serum creatinine was significantly elevated in the vehicle group, and there was a trend for lower levels in C23-treated mice (C23 v. vehicle, *p* = 0.052). (**B**) The BUN was significantly elevated in the vehicle group, and significantly reduced in C23-treated mice. (**C**) NGAL gene expression was significantly elevated in the vehicle group, and significantly reduced in C23-treated mice. (**D**) KIM-1 gene expression was significantly elevated in the vehicle group, and significantly reduced in C23-treated mice. *Data are presented as means* ± *SE*. *Number of animals in each group: sham n* = *5*, *vehicle n* = *7*, *C23 n* = *6*. *p < *0.05* *vs*. *sham*, ^#^*p* < *0.05* *vs*. *vehicle; one-way ANOVA followed by Student-Newman-Keuls test*.

Sepsis is also often associated with AKI. Therefore, we evaluated whether the effects of C23 on the development of sepsis-associated AKI by assessing renal filtration (Cr) and tubular excretion (BUN) tests at 20 h after CLP. Serum levels of Cr and BUN were, respectively 3.6- 3.8-fold higher in vehicle-treated mice than in sham-operated mice (Fig. 6A,B). Compared with vehicle, treatment with C23 significantly reduced BUN levels by 26%, and showed a trend towards lower Cr levels as well (Fig. 6A,B). Further evaluation showed that the renal mRNA expression of NGAL and KIM-1 was increased by 305.9- and 53.2-fold in vehicle-treated mice compared with sham-operated mice (Fig. 6C,D). The renal expression of renal injury markers NGAL and KIM-1, however, was reduced by 50% and 86% in mice treated with C23 as opposed to vehicle (Fig. 6C,D). Collectively, these data show that C23 is able to attenuate sepsis-induced AKI.

Next, we assessed proinflammatory cytokine levels in kidneys collected 20 h after CLP. The kidney levels of IL-6 were 2.9-fold higher in vehicle-treated mice than in sham-operated mice (Table 2). Conversely, kidney levels of IL-6 were 41.2% lower in C23-treated mice than in vehicle-treated mice (Table 2). Kidney levels of TNF-α and IL-1β were also increased in vehicle-treated mice (2.9- and 2.6-fold, respectively), and there was a statistical trend for lower kidney levels of TNF-α and IL-1β in C23-treated mice (Table 2). These data indicate that C23 is able to decrease local kidney inflammation caused by sepsis.

### C23 improves the survival after CLP

**Figure 7:**
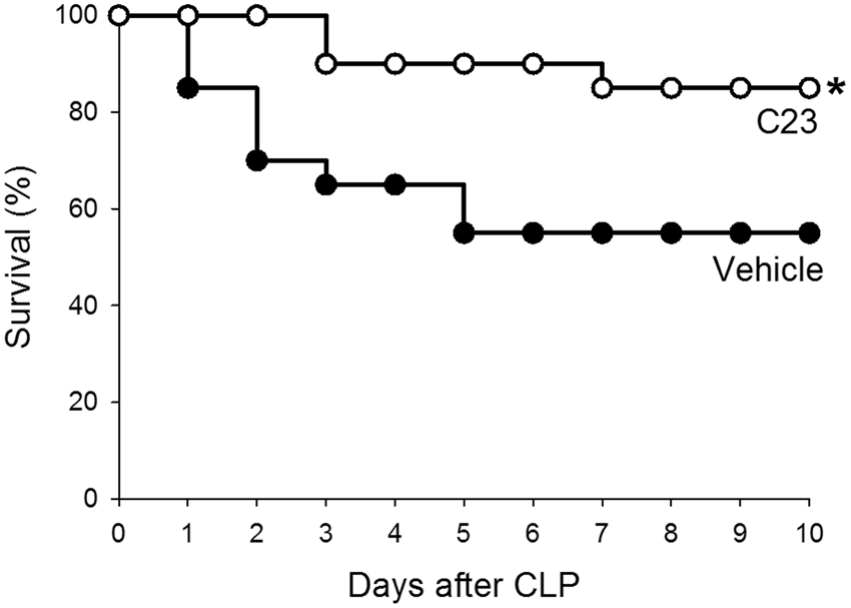
C23 improves survival after CLP. Mice subjected to CLP and treated with a single dose of vehicle or C23 at 2 h after CLP were observed for 10 days. Mice treated with vehicle had a 10-day survival of 55%, while mice treated with C23 had a significantly increased 10-day survival of 85%. *n* = *20* *per group*. *p < *0.05; Kaplan-Meier with log rank test*.

To determine whether C23’s observed attenuation of systemic, pulmonary, and renal injury and inflammation can improve the survival of septic mice, we induced CLP, treated mice with a single injection of vehicle or C23 at 2 h after CLP and observed the mice for survival over 10 days. Mice treated with vehicle had a 10-day CLP survival of 55% (Fig. 7). Mice treated with C23, on the other hand, had a 10-day CLP survival of 85% (Fig. 7). These observations indicate that C23 improves the survival after CLP.

## Discussion

In the last two decades, a large number of clinical trials have conducted in attempt to improve sepsis treatment. In spite of this intense search, effective anti-sepsis therapeutic strategies beyond antibiotics and supportive care are yet to be approved by the FDA. We have recently discovered that extracellular CIRP is a key mediator of injury and inflammation in preclinical models of sepsis and shock^18^. Serum levels of CIRP were further found to be elevated in septic patients and to be predictive of sepsis severity (APACHE II and SOFA scores), renal injury, and overall mortality^19^. Accordingly, we have hypothesized that CIRP inhibition would attenuate sepsis severity and mortality. Indeed, in the present study, we observed that sepsis treatment with the CIRP inhibitor C23 reduced systemic, pulmonary, and renal injury and inflammation.

CIRP is a 172-amino acid nuclear protein which regulates RNA translation^33–35^. CIRP is constitutively expressed in most tissues at low levels^33,36,37^. Cellular stressors such as hypothermia^15,38^, hypoxia^14^, oxidative stress^16^, and radiation^34,39–41^, upregulate CIRP expression, induce its translocation from the nucleus to the cytosol, promote its uptake by stress granules, and cause its release^16,34^. Once in the extracellular space, CIRP promotes the activation of vascular endothelial cells and the release of proinflammatory mediators by macrophages^18,20,42,43^. Indeed, rats treated with CIRP-neutralizing antibodies and mice genetically deficient in CIRP have decreased injury, decreased inflammation, and increased survival after CLP^13,18^. We have also discovered that extracellular CIRP plays a critical role in the pathogenesis of lung injury. The lungs of healthy mice injected with rmCIRP have high levels of proinflammatory cytokines, alveolar wall edema and neutrophil infiltration^20^.

Using surface plasmon resonance analysis, we have discovered that CIRP binds with high affinity for the TLR4-MD2 receptor complex^18,29^. After screening 32 oligopeptides (15-mers) covering the entire sequence of human CIRP for their affinities for the TLR4/MD2 complex, we identified C23 as a potential CIRP antagonist^18^. C23’s affinity for the TLR4/MD2 complex was one order of magnitude higher than that of CIRP, and two orders of magnitude higher than that of LPS or HMGB1^29^. Indeed, pre-incubation with C23 prevented rmCIRP-induced release of TNF-α from the human monocyte THP-1 cell line^18^, confirming that C23 is able to inhibit CIRP’s activity. We here showed that C23 inhibits CIRP-induced TNF-α release (dose-dependently), IκBα degradation, and NF-κB nuclear translocation. These results further support C23 ability to suppress extracellular CIRP-induced inflammation. These observations, along with the decreased severity and improved survival rate in septic mice treated with C23, suggest that targeting extracellular CIRP is a rational and feasible approach to treat septic patients.

We observed attenuation of systemic, pulmonary, and renal injury and inflammation in septic animals treated with C23. We chose to measure LDH, ALT, and IL-6, in particular because their serum levels are indicators of sepsis severity and mortality^44,45^. We also elected to investigate changes in lung tissue histology and levels of pro-inflammatory cytokines because they are crucially altered in sepsis-associated ALI^46,47^, and recapitulated by injection of CIRP into healthy mice^20^. In sepsis, EC apoptosis is increased and thought to contribute to lung injury^48,49^. We observed a reduced number of TUNEL-positive cells in the lungs of septic mice treated with C23. While intracellular expression of CIRP has been associated with cell survival^50–53^, we have correlated extracellular levels of CIRP with increased apoptosis in preclinical models of ALI, AKI, and hepatic ischemia and reperfusion^13,20,22,42^. The exact mechanism by which extracellular CIRP’s leads to apoptosis is poorly understood, although the DNA fragmentation detected using TUNEL may instead reflect pyroptosis, which is induced by extracellular CIRP via Nlrp3 inflammasome^20^. In fact, C23 attenuation of the inflammasome may have also directly contributed to lower serum and tissue levels of IL-1β^20^. In this study, C23 also decreased the levels of creatinine, BUN, NGAL, and KIM-1, all of which are increased during sepsis-associated AKI^54–57^. Considering the similarities between AKI caused by sepsis and ischemia and reperfusion, the beneficial effect of CIRP inhibition with C23 on sepsis-induced AKI is supported by our previous study that showed decreased AKI after renal ischemia and reperfusion in rats treated with CIRP-neutralizing antibodies and in CIRP-deficient mice^22^. Finally, we previously showed that CIRP causes EC activation and dysfunction^20^. Accordingly, in this study we observed lower expression of EC activation markers in the kidneys of mice treated with C23, corroborating our previous studies and indicating an important mechanism through which this peptide is able to attenuate sepsis severity.

C23’s postulated mechanism of CIRP inhibition is via competitive antagonism for the TLR4-MD2 receptor complex. Although it is conceivable that C23 might also displace the binding of other TLR4 agonists, we have not been able to inhibit the release of TNF-α after stimulation with LPS or HMGB1, suggesting that C23 binds to CIRP-specific binding sites in the TLR4-MD2 complex. Indeed, a computational modeling suggests that C23 displaces CIRP by docking into an MD2 pocket that is not a binding site for LPS (unpublished observations; W.-L. Yang and P. Wang). C23’s specificity for CIRP may be a pharmacological advantage, as TLR4 is a major player in the phagocytosis and clearance of bacterial pathogens^58–60^. Indeed, sepsis therapies completely blocking TLR4 have not been clinically successful^61,62^.

We have shown that LPS can cause CIRP release, and detected extracellular CIRP within 90 min of shock^18^, indicating that this mediator is in the circulation and able to promote disease severity since very early on in the course of sepsis. Therefore, in this study, we administered C23 at two h after CLP. Since elevated serum levels of CIRP are detected in septic patients within 24 h of admission to the intensive care unit^19^, administration of C23 at later, more clinically achievable time points (such as co-administration with the first dose of antibiotics) may also prove to be effective. C23’s half-life in the circulation is not known but, as an oligopeptide, it is expected to be short. Thus, it is possible that C23’s efficacy can be further increased by its administration in higher doses or by continuous infusion. Nonetheless, we here show that a single administration of C23 early in the course of sepsis is sufficient to generate enduring beneficial effects, as reflected by the survival study. C23’s lasting effect may be related to the inhibition of CIRP-induced release of TNF-α and HMGB1^18^.

This study has potential limitations that should be considered. C23’s pharmacokinetics and toxicology has never been studied, although significant side-effects are unlikely considering the benign phenotype of CIRP knockout mice^63,64^. The susceptibility and inflammatory response to CLP sepsis is sexually dimorphic in mice^25–28^. As female mice were not included in this study, the beneficial effects of C23 in females still need to be studied. By not including antibiotic therapy and analgesia, our model was more homogeneous but also more severe and departed from standards of care in sepsis. Since the PaO_2_/FiO_2_ ratio was not measured, we were unable to diagnose acute respiratory distress syndrome (ARDS) or to correlate the degree of respiratory shunt with lung histological injury and proinflammatory cytokines. TUNEL-positive cells were not further characterized and, therefore, we cannot determine their cell type or whether these cells are apoptotic or necroptotic. Finally, while the investigators were blinded only for the histology analysis, lack of blinding for other endpoints is a possible but unlikely source of bias, since all other measurements were determined directly and independently.

In conclusion, we have discovered that treatment with C23 attenuates lung and kidney injuries and inflammation, and improves the survival rate in mice with polymicrobial sepsis. Since CIRP can be released as a result of a number of insults, such as ischemia, hypothermia, and radiation, C23 has the potential to be beneficial for a plethora of diseases and conditions. Future studies should consider CIRP inhibition with C23 as a new potential therapeutic strategy to treat patients with sepsis and other conditions.

## Electronic supplementary material

Supplementary Figure 1
