# Supplementary material for: A cold-inducible RNA-binding protein (CIRP)-derived peptide attenuates inflammation and organ injury in septic mice
Source: Sci Rep. 2018 Feb 12;8:3052. doi: 10.1038/s41598-017-13139-z (PMC5809586; doi:10.1038/s41598-017-13139-z)
Supplement: Supplementary file 1 — Supplementary Figure 1 [file 41598_2017_13139_MOESM1_ESM.pdf]

A cold-inducible RNA-binding protein (CIRP)-derived peptide attenuates inflammation and organ injury in septic mice

Fangming Zhang [1], Max Brenner [1], Weng-Lang Yang [1,2], Ping Wang [1,2 *]

[1] *Center for Immunology and Inflammation, The Feinstein Institute for Medical Research, Manhasset, NY 11030;* [2] *Department of Surgery, Hofstra Northwell School of Medicine, Manhasset, NY 11030*

**** Corresponding author***

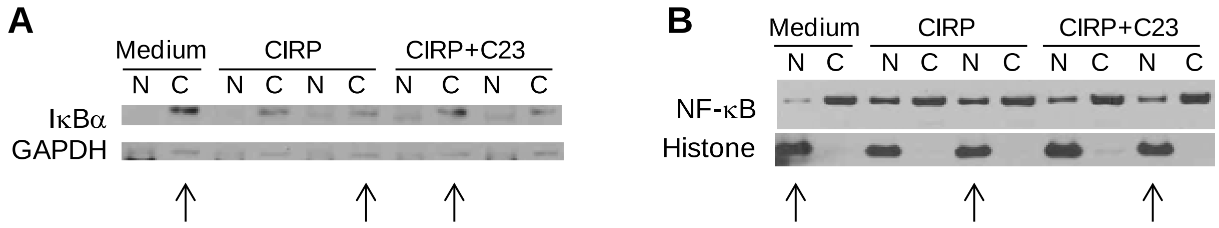

Supplementary Figure 1. C23 inhibits CIRP-induced NF-κB activation. Recombinant mouse (rm) CIRP promoted (**A**) degradation of cytosolic IκBα and (**B**) nuclear translocation of NF-κB p65. These effects were partially reverted by C23. Cultured RAW 264.7 cells were co-incubated with rmCIRP (300ng/ml) in the absence or presence of C23 for 4 h, fractionated into nuclear (N) and cytoplasmic (C) extracts, and immunoblotted using antibodies for IκBα and GAPDH, or NF-κB p65 and histone. Arrows indicate the blots presented in Figure 1.
